# Exploring prevalence of risky sexual behavior among adolescents in selected high schools of Bulawayo, Zimbabwe: a mixed-method study protocol

**DOI:** 10.3389/frph.2025.1744889

**Published:** 2025-12-19

**Authors:** Refiloi Ndlovu, Perez Livias Moyo

**Affiliations:** Department of Environmental Health, Faculty of Environmental Science, National University of Science and Technology, Bulawayo, Zimbabwe

**Keywords:** risky sexual behaviour, adolescents, sexual health, high schools, Bulawayo

## Abstract

**Introduction:**

Risky sexual behaviour (RSB) among adolescents in Zimbabwe is a major public health issue, contributing to high rates of HIV, STIs, and unintended pregnancies. Interventions have been ineffective, especially in urban areas like Bulawayo, where “Vuzu parties” worsen the situation. This study aims to assess the prevalence of RSB among high school students in Bulawayo, identify influencing factors, evaluate awareness of sexual health issues, and examine the accessibility and effectiveness of sexual health education from various stakeholders’ perspectives.

**Methods:**

The primary study is a school-based, convergent mixed-methods design that includes a quantitative cross-sectional survey and a qualitative key informant study, conducted concurrently. A preliminary systematic review will inform the study's framework and instrument development. The quantitative phase will survey 400 students, selected through stratified random sampling from five schools in Bulawayo, using a self-administered questionnaire. The qualitative phase will consist of in-depth interviews with 10–15 teachers and healthcare workers. Quantitative data will be analysed with descriptive statistics and chi-square tests in SPSS version 28, while qualitative data will undergo thematic analysis. A joint display table will integrate the findings to identify areas of convergence, divergence, and complementarity.

**Discussion:**

The study will provide comprehensive data on the prevalence and drivers of RSB in Bulawayo's adolescents. Findings will inform the development of targeted school-based interventions and policies aimed at reducing RSB and improving sexual health outcomes. The study findings will be reported back to the public and health officials through community meetings and workshops.

## 1 Introduction

Adolescence is a crucial period in human development (1) It can also be defined as a time when a person transitions from childhood to adulthood and experiences major changes in their biology, psychology, and social interactions (2). According to the WHO, adolescents are individuals in the 10–19-year age group (1, 3, 4). This means that all the students in high schools are adolescents. Young people at this stage are often tempted to engage in sexual activities. Sexual risk behaviors refer to any sexual activity that could increase a person's chance of contracting HIV or other STDs, as well as an unexpected pregnancy (5).

Some scholars consider behaviors associated with sexually transmitted infections (STIs), unprotected sex, early sexual debut, and unexpected pregnancy as risky sexual behavior (6). According to the NHS, unprotected sex imposes a risk of pregnancy and contracting or spreading STIs, which include chlamydia, genital herpes, genital warts, gonorrhea, HIV and syphilis. Anal sex is also listed as a risky sexual behavior because the lining of the anus is thin and can easily be damaged, which makes it more vulnerable to infection. Oral sex is another risky sexual behavior listed by the NHS (7). There is a high chance of getting infections when you have sores or cuts around the mouth, genitals or anus. Giving your partner oral sex when you are sick could expose them to the herpes virus. Herpes can also spread from the genitalia to the oral cavity (8, 9).

Teenage sexual activity is a global concern, and many secondary schools (where the majority of adolescents attend) find it difficult to regulate this risky sexual behavior (10). According to the WHO, 11% of births globally and 23% of the disease burden in Disability Adjusted Life Years (DALYs) from pregnancy and childbirth among women of all ages are caused by teenage pregnancies (11).Worldwide, women under the age of 20 give birth to 13 million children, with more than 90% of these births occurring in poor nations (11).

In a survey carried out in the United States, it was observed that numerous high school students participate in sexually risky activities that increase their chance of contracting STIs, including HIV infection, and unplanned pregnancies. 39.5% of students nationwide reported having had sex at some point in their lives, while 9.7% reported having sex with four or more people (12). Tanzania has also experienced an increase in school dropouts due to pregnancy, with an estimated number of 28,600 girls leaving school due to pregnancy (13). In a survey carried out in Nigeria, it was discovered that teenagers are known for their risky sexual behaviors, which include multiple sexual partners, risky sexual behavior, and early sexual activity. Among the justifications offered for this are financial gain, enjoyment, peer pressure, curiosity, and others (14).

In South Africa it was discovered that between 50% and 60% of young people who are sexually active report never using condoms, the majority of school students who had ever had sex reported having no more than one partner in the previous year, with a persistent minority of between 1% and 5% of females and 10%–25% of males having more than four partners annually (15). In Zimbabwe's secondary schools, the number of school-age teenagers who participate in sexually risky behavior is clearly significant and is said to be more prevalent in urban (10). Adolescents at rural secondary schools are no longer exempt from this issue, which was previously assumed to be more noticeable in urban secondary schools, where cultural morals are thought to be less rigid due to the dissolution of traditional family structures and the spread of media technology (16).

In Bulawayo, teenage risk-taking is a big problem. Newspapers have been reporting on students secretly attending parties where 13-year-old girls are crowned for sleeping with more than ten men in one night, while boys receive US dollar notes for sleeping with the most girls in one event (16). These parties are known as Vuzu parties, and it is at these parties that teenagers abuse various drugs that are harmful to their health and engage in other risky and unacceptable behaviors (17). It is concerning that these activities are increasing despite efforts by the government and other stakeholders to launch massive sensitization programs and awareness campaigns to conscientize youths about the pitfalls associated with such behaviors. In fact, numerous programmatic interventions have been implemented to sensitize and raise awareness among these youths about the dire consequences of their actions, but the trend continues. However, there is limited research specifically addressing risky sexual behaviors among adolescents in Bulawayo, Zimbabwe. This study aims to comprehensively investigate the prevalence and awareness of risky sexual behavior among the students in Bulawayo using a mixed-methods approach.

## 2 Methodology

### 2.1 Research approach

This study will employ a convergent mixed-methods design. This design involves collecting and analysing quantitative and qualitative data separately but concurrently, with the integration of findings occurring during the interpretation phase. The rationale for this design is that the combination of both datasets provides a more complete understanding of the research problem than either approach alone. The quantitative component (cross-sectional survey) will provide broad, generalisable data on the prevalence and correlates of RSB. The qualitative component (key informant interviews) will provide detailed, contextual insights into the mechanisms, perceptions, and systemic factors influencing RSB. A preliminary systematic review will be conducted to synthesise existing global evidence, which will inform the development of the survey instrument and interview guides for the primary mixed-methods study, ensuring they are grounded in established evidence.

### 2.2 Phase 1: preliminary literature review

This review is conducted to establish a comprehensive theoretical and contextual framework for the primary mixed-methods study. Its findings will directly inform the domains and specific questions included in the quantitative survey and qualitative interview guides.

#### 2.2.1 Review title

Risky sexual behaviour among adolescents: a systematic literature review.

#### 2.2.2 Objectives

The main objective of the systematic review is to provide evidence-based understanding of risky sexual behaviour in adolescents, including its prevalence, risk factors, consequences, and useful interventions globally, to guide the development and focus of the primary data collection instruments and analysis. The specific objectives of this study are listed in Table 1.

**Table 1:** Research summary.

Study phase	Objectives	Data sources & collection	Data management & analysis	Integration function
Preliminary systematic review	1. To assess the global prevalence of RSB among adolescents. 2. To identify factors influencing RSB. 3. To analyse the consequences of RSB. 4. To review interventions aimed at reducing RSB.	**Sources:** Electronic Databases (Google Scholar, ScienceDirect, PubMed, Cochrane Library, Web of Science). **Collection:** Document retrieval for studies published between 2008 and 2024.	**Screening:** Titles/abstracts screened against eligibility criteria. **Extraction:** Data extracted using a standardised form. **Appraisal:** Quality assessed using JBI checklists. **Synthesis:** Narrative summary & thematic tables.	Informs the development of the quantitative survey and qualitative interview guide for the primary study.
Quantitative survey	1. To determine the prevalence of RSB among adolescents in Bulawayo. 2. To identify factors leading to RSB. 3. To determine awareness levels of sexual health & HIV/AIDS.	**Sources:** 400 students from 5 selected schools. **Collection:** Self-administered questionnaire via Google Forms. **Sampling:** Stratified random sampling.	**Software:** SPSS Statistics v.28. **Analysis:** Descriptive statistics (frequencies, percentages); Chi-square tests to determine associations between variables. **Presentation:** Tables & graphs.	Provides numerical data for integration. Preliminary results will be used to refine qualitative probing. Data is merged with qualitative findings during interpretation.
Qualitative study	1. To investigate the accessibility of sexual health education for adolescents. 2. To explore the effectiveness of this education. 3. To assess stakeholder attitudes towards it.	**Sources:** Purposively sampled key informants (teachers, healthcare workers). **Collection:** Semi-structured interviews using a guided protocol.	**Processing:** Interviews transcribed verbatim. **Analysis:** Thematic Analysis following the framework by (20) to identify, analyse, and report key themes.	Provides contextual depth for integration. Explores and explains the quantitative findings. Data is merged with quantitative findings during interpretation
Data integration	To synthesise quantitative and qualitative findings to develop a comprehensive understanding of RSB in Bulawayo.	**Sources:** Outputs from the Quantitative Survey and Qualitative Study.	**Analysis:** Development of a joint display table to compare and contrast findings. Identification of points of convergence, complementarity, and divergence.	A distinct phase where the two datasets are formally brought together to answer the research questions holistically.

#### 2.2.3 Methodology

##### 2.2.3.1 Eligibility criteria (PICOS)

- Population: Adolescents (aged 10–19 years).
- Interest/Exposure: Risky sexual behaviour (e.g., unprotected sex, multiple partners, early sexual debut).
- Comparator: Not applicable for this prevalence review.
- Outcomes: Prevalence rates, influencing factors, consequences (STIs, HIV, pregnancy), and intervention strategies.
- Study Designs: Quantitative, qualitative, and mixed-methods studies published in peer-reviewed journals.

Inclusion criteria: Studies published in English between January 2008 and December 2024 that focus on adolescents and directly address RSB.

Exclusion criteria: Studies focusing on adults or children outside the adolescent age range, non-English publications, and commentaries or editorials without original data.

###### 2.2.3.1.1 Search strategy

Electronic databases (Web of Science, PubMed, ScienceDirect, Cochrane Library) will be searched using the keywords: (“risky sexual behaviour” OR “risky sexual behaviour”) AND (adolescent OR teen* OR “high school student”) *. The search will be limited to the specified date range. A snowballing technique will be used to identify additional relevant articles from reference lists.

###### 2.2.3.1.2 Study selection and data extraction

Titles and abstracts will be screened independently by the researchers against the eligibility criteria. Full-text articles of eligible studies will be retrieved and reviewed. Data will be extracted using a standardised form capturing author, year, country, sample size, methodology, key findings on prevalence, factors, and outcomes.

##### 2.2.3.2 Quality assessment

The quality of included studies will be assessed using the Joanna Briggs Institute (JBI) critical appraisal checklists for prevalence studies.

##### 2.2.3.3 Data synthesis

Extracted data will be summarised narratively and presented in tables to illustrate the prevalence, factors, and consequences of RSB across different contexts.

### 2.3 Phase 2: primary mixed-methods study

#### 2.3.1 Study setting

The study will be conducted in Bulawayo, the second-largest city in Zimbabwe, with an estimated population of 665,952. The adolescent population is estimated at 186,265 females and 182,330 males (18). The city has 41 registered secondary schools. The study will purposively select one school from each of the city's five administrative districts to ensure geographical representation.

#### 2.3.2 Quantitative component: cross-sectional survey

##### 2.3.2.1 Study population and sampling

The target population is all adolescents enrolled in high schools in Bulawayo (estimated *N* = 368,595). The sample size will be calculated using a population size of 368,595, a 95% confidence level, a 5% margin of error, and a response distribution of 50%, resulting in a minimum sample size of 384. To account for non-response and attrition, the sample size will be rounded up to 400 students. One school will be purposively selected from each of the city's five districts, and 80 students will be randomly chosen from each school using stratified random sampling (by form level) to ensure grade and gender representation.

Inclusion criteria: All adolescents aged 10–19 enrolled in the selected schools who provide assent and have parental/guardian consent.

Exclusion criteria: Adolescents not enrolled in formal school, those unwilling to participate, or those without required consent.

###### 2.3.2.1.1 Data collection methods and tools

A structured, self-administered questionnaire will be developed for this study based on the findings of the preliminary systematic review and a review of established instruments, such as the Youth Risk Behaviour Survey by the Centres for Disease Control and Prevention (19), to ensure comprehensive coverage of socio-demographics, RSB prevalence, knowledge, and attitudes. The tool will undergo a rigorous validation process, including expert review by a panel of public health and methodology specialists to establish content and face validity, followed by pre-testing and cognitive interviews with a small group of adolescents to enhance clarity and cultural appropriateness. Finally, a pilot study will be conducted to assess the internal consistency reliability of scaled sections using Cronbach's alpha. The finalised questionnaire will be administered via Google Forms on tablets in school computer labs to ensure privacy and data security.

###### 2.3.2.1.2 Data analysis

Data will be extracted from Google Forms into Microsoft Excel for cleaning and then imported into SPSS version 28 for analysis. Descriptive statistics (frequencies, percentages, means) will be used to summarise socio-demographic characteristics and the prevalence of RSB. Inferential statistics, primarily Chi-square tests, will be used to determine associations between demographic factors and the prevalence of RSB. Findings will be presented in tables and graphs.

#### 2.3.3 Qualitative component: key informant interviews

##### 2.3.3.1 Study population and sampling

Key informants will be purposively selected to include individuals with direct professional engagement with adolescent sexual health. This will include approximately 10–15 participants: life skills teachers, school counsellors, school nurses, and local community health workers from the selected schools and their surrounding clinics. The approximated sample size will be guided by data saturation, meaning no new themes or information will be emerging from the data, so researchers will continue data collection until this point is confirmed.

###### 2.3.3.1.1 Data collection methods and tools

Semi-structured interviews will be conducted using a pre-tested interview guide. The guide will be developed based on initial findings from the systematic review and emerging questions from the quantitative survey analysis to allow for probing and exploration of the quantitative results. The guide will explore themes such as the perceived prevalence of RSB, accessibility of sexual health education and services in schools, challenges faced, and recommendations for improvement. Interviews will be conducted in a private setting, audio-recorded with permission, and supplemented with field notes.

###### 2.3.3.1.2 Data analysis

Audio recordings will be transcribed verbatim. Thematic analysis, following the six-step framework by (20) will be employed. This involves familiarisation with the data, generating initial codes, searching for themes, reviewing themes, defining and naming themes, and producing the report. Trustworthiness will be ensured through peer debriefing and member checking. Direct quotes will be used to illustrate the themes.

#### 2.3.4 Integration of quantitative and qualitative data (triangulation)

Integration will occur at two stages:

##### 2.3.4.1 During data collection

The initial qualitative interview guide will be informed by the systematic review of literature. As quantitative data is analysed, preliminary findings (e.g., surprising prevalence rates, significant associations) will be used to refine the interview guide and probe deeper into these areas during subsequent qualitative interviews.

##### 2.3.4.2 During data interpretation

Following a separate analysis of the quantitative and qualitative datasets, the findings will be integrated using a joint display table. This table will juxtapose quantitative results (e.g., “X% of students reported early sexual debut”) with related qualitative findings (e.g., “Teachers attributed early sexual debut to peer pressure at social gatherings like Vuzu parties”). This side-by-side comparison will allow for the identification of:

- Convergence: Where the two datasets confirm or reinforce the same conclusion.
- Complementarity: Where the two datasets elucidate different facets of a phenomenon, providing a richer, more nuanced picture.
- Divergence/Contradiction: Where the two datasets present conflicting findings, which will be explored as valuable insights into the complexity of the issue.

### 2.4 Proposed timelines

This research will be conducted from January 2026 to November 2026. Development and piloting of data collection tools will be conducted from January to February. The actual data collection will then be carried out in March and May. Data entry and analysis will be done from June to July. Lastly, manuscript writing and submission to a reputable peer-reviewed journal will be done from August to November 2026.

## 3 Discussion

The convergent mixed-methods design is a significant strength of this study. The inclusion of a preliminary systematic review ensures the primary study is grounded in existing global evidence. The planned systematic review aims to synthesize a significant amount of global evidence, which is expected to yield several key findings. It is anticipated that the review will reveal considerable variation in the prevalence of risk-taking behaviors (RTB) among adolescents across different cultural and socioeconomic contexts. At the same time, it will likely identify a core set of influencing factors, including peer pressure, lack of parental supervision, socioeconomic disadvantage, substance use, and gaps in comprehensive sexual knowledge. The review is expected to highlight the severe consequences of these risk behaviors, particularly the increased risk of HIV and other sexually transmitted infections (STIs), unintended teenage pregnancies, and subsequent school dropouts. A critical outcome of the review will be the identification of effective intervention strategies, which may include school-based comprehensive sex education, youth-friendly health services, and community engagement programs. The synthesized evidence from this review will directly inform the development of the data collection instruments for the primary study, ensuring that both the quantitative survey and qualitative interview guides are based on global evidence tailored to investigate the most relevant factors within the context of Bulawayo.

In addition to its methodological contributions, the findings of this study are expected to have significant practical implications for public health policy and school-based programs in Bulawayo. The quantitative data will offer the first detailed, school-level overview of RSB prevalence in the city, advancing beyond anecdotal media coverage to provide evidence that can inform targeted interventions. For example, if the data indicate specific age groups (such as early adolescents in Form 1) or demographic subgroups with elevated risk profiles, resources can be allocated more effectively. Furthermore, the qualitative insights gathered from teachers and healthcare workers will shed light on the systemic and socio-cultural barriers that impede effective sexual health education, including curriculum deficiencies, cultural taboos, and insufficient teacher training.

This study aims to contribute significant theoretical insights into the ecosystem of adolescent risk behavior within the complex urban context of Africa. The phenomenon of “Vuzu parties” highlights a multifaceted interplay of factors such as poverty, peer pressure, gender dynamics, and identity exploration elements that may elude traditional behavioral models. Integrating quantitative data on prevalence rates with qualitative narratives that elucidate the perceived benefits and social pressures associated with these gatherings, we can construct a theoretical framework for understanding risk-seeking behavior in Bulawayo. Such a framework has the potential to inform the design of culturally relevant behavior-change communication strategies and peer education programs that directly address the local determinants of risky sexual behaviours.

We recognize that the study's cross-sectional design, while essential for establishing baseline data, restricts our ability to make causal inferences. Consequently, our discussion of the integrated findings will thoughtfully frame the observed associations within this limitation, avoiding assertions of causality. Instead, we will focus on the temporal and contextual relationships identified. Additionally, we will explicitly address the transferability of our findings. While the focus on in-school settings may limit generalization to all adolescents, the rich contextual details from our qualitative data will enable policymakers and practitioners in similar environments to assess the applicability of our results to their specific contexts.

## 4 Ethics and dissemination

Ethical approval for this study was obtained from the National University of Science and Technology Institutional Review Board (Ref: NUST/IRB/2025/75). Given the sensitive nature of the research, several key ethical and safety measures will be implemented. For the quantitative survey, written informed consent will be obtained from parents or guardians, and written assent will be secured from all adolescent participants. For the qualitative interviews, informed consent will be obtained from all key informants. Anonymity and confidentiality will be rigorously maintained; no personally identifiable information will be collected in the questionnaires, and all data will be stored on password-protected computers. Participants will be informed of their right to withdraw at any time without penalty. To mitigate potential distress, a list of local counselling and support services will be provided to all participants.

This study aims to share its findings in various ways to effectively reach different audiences, including academics, policymakers, and community members. The main results will be submitted for publication in a recognised international journal focused on public health. Additionally, important findings will be shared during community meetings and workshops. A summary report, crafted in plain language, will be provided to the Bulawayo Provincial Education Office, the Ministry of Health and Child Care, and the participating schools. This approach is intended to guide local policy decisions and intervention strategies. De-identified quantitative dataset will be deposited in a public data repository (Zenodo) upon study completion to ensure data curation and availability for future research.

## 5 Conclusion

The anticipated findings from this study are expected to have significant implications. They are likely to validate concerns highlighted by media reports and preliminary studies about activities such as Vuzu parties, thereby providing concrete evidence to inform public health responses. Additionally, the results will play a crucial role in assessing the current state of sexual health education in schools, pinpointing gaps in accessibility, content, and effectiveness. Ultimately, the integrated evidence from all three phases is intended to establish a solid foundation for evidence-based interventions. These could encompass targeted peer-education programs, revised school curricula that address specific identified risk factors (e.g., substance abuse, peer pressure), and community engagement initiatives designed to counteract the normalisation of risky behaviours.

## 6 Limitations

This study protocol acknowledges several potential limitations despite its comprehensive design. Firstly, the cross-sectional nature of both the quantitative and qualitative components means that the data only provide a snapshot in time, which inhibits the ability to establish causal relationships between variables. Additionally, the focus of the study on in-school adolescents excludes out-of-school youth, who may be at an even greater risk of engaging in risky sexual behaviours, potentially resulting in an underestimation of the true prevalence within the broader adolescent population. Finally, while the mixed-methods design is a strength, the success of integration depends on the researcher's interpretative rigour; we will mitigate this through peer debriefing during the analysis phase.
